# Enhanced Multi-Class Breast Cancer Classification from Whole-Slide Histopathology Images Using a Proposed Deep Learning Model

**DOI:** 10.3390/diagnostics15050582

**Published:** 2025-02-27

**Authors:** Adnan Rafiq, Arfan Jaffar, Ghazanfar Latif, Sohail Masood, Sherif E. Abdelhamid

**Affiliations:** 1 Department of Computer Science & IT, Superior University, Lahore 54000, Pakistanarfan.jaffar@superior.edu.pk (A.J.); sohailmasood@superior.edu.pk (S.M.); 2 Department of Computing Science, Thompson Rivers University, Kamloops, BC V2C 0C8, Canada; 3 Department of Computer Science, Prince Mohammad Bin Fahd University, Al-Khobar 34754, Saudi Arabia; 4 Department of Computer and Information Sciences, Virginia Military Institute, Lexington, VA 24450, USA; abdelhamidse@vmi.edu

**Keywords:** breast cancer classification, deep learning, deep features, deep neural network, densenet121, histopathology images, different magnification levels

## Abstract

**Background/Objectives:** Breast cancer is among the most frequently diagnosed cancers and leading cause of mortality worldwide. The accurate classification of breast cancer from the histology photographs is very important for the diagnosis and effective treatment planning. **Methods**: In this article, we propose a DenseNet121-based deep learning model for breast cancer detection and multi-class classification. The experiments were performed using whole-slide histopathology images collected from the BreakHis dataset. **Results**: The proposed method attained state-of-the-art performance with a 98.50% accuracy and an AUC of 0.98 for the binary classification. In multi-class classification, it obtained competitive results with 92.50% accuracy and an AUC of 0.94. **Conclusions**: The proposed model outperforms state-of-the-art methods in distinguishing between benign and malignant tumors as well as in classifying specific malignancy subtypes. This study highlights the potential of deep learning in breast cancer diagnosis and establishes the foundation for developing advanced diagnostic tools.

## 1. Introduction

Especially for those between the ages between 30 and 69 years, the “2020 World Cancer Report” by the International Agency for Research on Cancer (IARC) highlights cancer as one of the leading causes of death globally in 134 out of 183 countries [1]. Lung cancer ranks as the most regularly occurring cause of cancer-related death in men and women. Prostate cancer is more common in men, but breast cancer (BC) leads among women. IARC projects that cancer diagnoses will climb dramatically from 18.1 million in 2018 to 29.5 million in 2040, and cancer-related fatalities from 9.6 million to 16.4 million within the same period [2]. Among the most often occurring and lethal types of cancer affecting women all around is breast cancer. Affecting around 2.1 million women annually, it makes for almost 14% of all cancer cases reported. For 2020 alone, BC claimed a projected 665,000 deaths worldwide. The high death rates found in developed countries are also rising everywhere.

Whether benign (non-cancerous) or malignant (cancerous), unusual cell division produces a development known as a tumor. Usually beginning with milk-producing lobules or ducts carrying milk to the nipple, breast cancer is a malignant tumor developing in breast tissue [3]. The tumor could finally affect other body regions by spreading to surrounding lymph nodes and tissues. With 85–90% of instances connected to genetic changes brought on by ageing and environmental factors, breast cancer stages define the degree of its spread [4]. Although the exact reasons for BC are unknown, DNA damage or mutations inside cells are considered rather important. The three main forms of BC are “invasive carcinoma”, “ductal carcinoma”, and “invasive lobular carcinoma” [5]. The most often occurring invasive carcinoma comes from milk ducts and travels to surrounding tissues. A subset of invasive carcinoma, invasive lobular carcinoma, grows in milk glands [6].

Early-stage breast cancer detection performed effectively significantly reduces tissue damage and raises the possibility of recovery. Clinically, magnetic resonance imaging (MRI), mammography, histology, and ultrasonic waves are several methods used. Among these, MRI is sometimes used because of its improved sensitivity in spotting anomalies relative to other screening techniques [7]. Conversely, histopathology provides important predictive information that can help to pinpoint important targets early in the diagnostic process. In histological techniques, a tissue sample is taken from the afflicted area and examined under a microscope to ascertain whether the tumor is benign, malignant, or expected [8]. Usually, the low magnification levels of histological pictures allow for thorough tissue-level investigation. Cancerous cells can be evaluated using criteria covering cell form, size, nuclear shape, geographic distribution inside the tissue, etc. Treatment planning, especially in determining the tumor size before surgical intervention, mostly depends on histological evaluations. This approach improves patient outcomes and guarantees proper therapy strategies [9].

With advancements in machine learning frameworks [10,11,12,13,14] and imaging technologies [15,16], digital histopathology has become a standard, cost-effective, and reliable cancer diagnosis method. Cancer cells infiltrate the basement membrane of ductal–lobular structures in breast tumors and spread into surrounding tissues [17]. Various algorithms have been developed to classify breast histopathology images by analyzing features such as spatial distribution and frequency domain morphology [18]. Data-driven methods and deep learning approaches, especially convolutional neural networks (CNNs), have been extensively used in recent decades to classify images of breast cancer histology [19]. Still, there are difficulties in reaching self-interpretability in diagnosis techniques, which could cause mistakes.

Manual histological examination is a traditional diagnostic method that requires time and is prone to subjective interpretation errors due to inter-observer variability. Considering the rapid development in digital histology and imaging technologies, automated approaches have been studied to increase the dependability and efficiency of breast cancer diagnosis. Different current approaches for breast cancer classification suffer from issues like inadequate generalization over different datasets, poor interpretability, and insufficient feature extraction. Although convolutional neural networks (CNNs) and deep learning models have shown promise in this field, a strong, efficient, and very accurate model able to overcome these constraints is still much needed. The major contributions of our study are as follows:

- We propose a deep learning network derived from DenseNet121 for the histological image categorization of breast cancer. The tightly coupled design provides effective feature extraction and accelerated performance by permitting feature reuse and, hence, lowering the vanishing gradient problem.
- Our study focuses on effectively utilizing a single, widely recognized dataset, demonstrating how advanced deep learning techniques can achieve high classification accuracy without needing multiple datasets. This simplifies the implementation while maintaining reliable performance.
- The model is explicitly fine-tuned for classification tasks, achieving robust and accurate predictions. This makes it a practical solution for breast cancer diagnosis in histopathological image analysis.

Section 2 analyzes present approaches, including machine learning and deep learning techniques, and highlights their shortcomings, such as reliance on many datasets, inefficiencies in feature extraction, or lack of generalization. The suggested method handles the used dataset, preparation techniques, DenseNet121 architecture, and training methods, including hyperparameter settings and assessment criteria. The experimental findings expose the model’s performance in classification tasks, along with a comparative study with previous methods and images, such as confusion matrices and learning curves containing accuracy, precision, recall, and F1-score. We address the strengths and limitations of the work with an eye toward the advantages of using a single dataset and the prospects for generalization in breast cancer classification. Finally, Section 5 lists the contributions, underlining the model’s success and suggesting future improvements, such as enlarging to other datasets or enhancing interpretability. The paper ends with a thorough References Section referencing pertinent tools and studies.

## 2. Related Work

Deep learning methods, especially convolutional neural networks (CNNs), have shown great potential in automating the interpretation of medical images, including mammograms, ultrasounds, and histopathology slides in categorizing breast cancer. These approaches eliminate the requirement for hand feature extraction by learning hierarchical features from raw pixel data using big, annotated datasets. Recently, CNN designs, including ResNet, Inception, and U-Net, have improved tumor detection and classification accuracy and efficiency. Early identification has shown great promise for these methods, which also enable radiologists to more precisely and with less variability diagnose breast cancer. This study reviews the most recent advances in deep learning for breast cancer image classification and presents an analysis of numerous models and a discussion of ongoing challenges in improving performance and generalization.

Attributed to its capacity to independently extract and categorize essential elements from complicated picture data, Mustafa et al. [20] highlighted CNNs as a dominating deep-learning method for cancer detection. Usually employing either creating models from scratch or using transfer learning with pre-trained networks, such as AlexNet, ResNet, GoogLeNet, or VGGNet, CNN development takes two paths. Adapting the last layers for binary and multi-class tasks, Yari et al. [21] classified breast cancer histomorphology images using ResNet50 and DenseNet-121. Images independent of magnification received 99.26% accuracy in their binary classification; images dependent on magnification obtained 99.02% to 100% accuracy in their binary classification. Transfer learning is successful in this domain since multi-class classification shows MD accuracies of 95.57% for ML classification and 94.95% to 97.96%.

Focusing on separating MD and MI categories, Boumaraf et al. [22] used the ResNet18 pre-trained model for binary and multi-class classification tasks. They used a block-wise method to fine-tune the model using the BreakHis dataset, which allowed them to enhance the classifier by incorporating an extra fully connected (FC) layer. Our technique achieved excellent image-level accuracies of 98.84% for binary MD classification and 92.15% for eight-class classification, whereas MI classification produced 98.42% and 92.51% for binary and eight-class tasks, respectively. Similarly, Sheikh et al. [23] presented MSI MFnet, a multi-scale input and multi-feature network to manage histological picture magnitudes. MSI MFnet successfully fused hierarchical feature maps to capture cellular characteristics and tissue texture. Applying the approach to the BreakHis dataset yielded patch-wise accuracies of 98% for binary classifications and 87% for multi-class classifications, while using it for the ICIAR2018 dataset yielded 82% and 68% accuracy, respectively. These results show that multi-scale input and feature fusion are valuable for improving classification performance.

Modern developments in attention processes have greatly improved the effectiveness of deep learning models in computer vision applications, including breast cancer categorization. These systems constantly allocate weights to prioritize pertinent elements, mimicking the human visual system. Togacar et al. [24] presented the CBAM-based BreastNet architecture in breast cancer histology, which processes feature maps across spatial and channel dimensions to increase accuracy and efficiency. Combining convolutional, dense, and residual blocks with a hyper-column approach, BreastNet achieves classification accuracy between 95.89% and 98.52%. Li et al. [25] modified the squeeze-and-excitation (SE) technique into DenseNet121 to create IDSnet, which uses global average pooling to lower complexity and minimize overfitting. Reaching 84.5% to 89.1% image recognition rates, IDSnet outperformed models, including ResNet50 and VGG-16. Zou et al. [26] presented AHoNet, an improved ResNet18 with an efficient channel attention (ECA) module and matrix power normalizing accuracy of up to 99.29% and 85%, respectively, obtaining remarkable results on BreakHis and BACH datasets.

Demir presented a convolutional-LSTM (CLSTM) deep learning model for automated breast cancer classification, combining marker-controlled watershed segmentation for pre-processing and an optimum Support Vector Machine (SVM) for decision-making [27]. Using the BreakHis dataset, the model produced accuracies of 94.91% (40×), 96.12% (100×), 95.51% (200×), and 95.42% (400×). Still, the approach relied on robust gear and processing capability. In an identical line, Wang et al. [28] developed the DBLCNN network, a deep learning model with magnification independence that modified MobileNet with transfer learning. This reduction in model parameters and calculation time reduced good recognition performance, even while it preserved it. Several recent papers have examined deep learning methods for breast cancer histomorphology photo-categorization using contemporary architectures and algorithms. Sharma and Kumar [29] obtained an adequate classification accuracy over several magnification levels (40× to 400×) using a radial basis function kernel on the pre-trained Xception model with an SVM classifier. The published BCHisto-Net, a CNN-based model with 89% accuracy that extracted global and local features to identify 100× magnification photos from the BreakHis collection, was presented by Rashmi et al. [30]. Combining synthetic labels based on clustering with transfer learning for histology data, Dif et al. [31] put forth a hybrid method. Other notable attempts are those of Wang et al. [32], who combined CNN features using manifold learning; Sanyal et al. [33], who created a hybrid ensemble network using fine-tuned CNNs and XGBoost (version 2.1.2) for feature extraction and classification; Zerouaoui and Idri [34], who also employed a hybrid architecture, including seven CNN models, using Borda count voting to find the optimal configuration. These techniques, together, demonstrate how well deep learning may improve breast cancer diagnosis.

## 3. Proposed Framework

**Figure 1:**
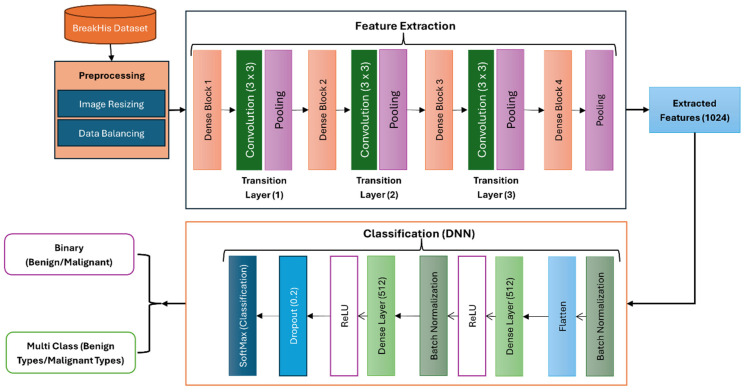
Proposed DenseNet121-based architecture for binary and multi-class breast cancer classification using image histopathology dataset.

The suggested approach features from breast cancer histology images using DenseNet121, a deep convolutional neural network, as the backbone. DenseNet121 was selected for its capacity to effectively use parameters and capture complex spatial and semantic aspects through dense connectedness. Preprocessing activities on the features obtained help to improve data quality and guarantee fitness with the following classifiers. Support vector machines (SVMs), Random Forests (RFs), XGboost, and a deep neural network (DNN) classifier, among other machine learning techniques, then take advantage of these characteristics. This hybrid method seeks to produce a strong and accurate classification by combining the adaptability and interpretability of conventional machine learning techniques with the feature-extracting capability of deep learning. The architecture of the proposed system is shown in Figure 1.

### 3.1. Preprocessing

First downsized for preparation to a constant dimension fit for deep-learning models, the breast cancer histopathology photos were then Resized to 224 × 224 pixels; the DenseNet121 model’s standard input size guarantees fitting with the pre-trained network. This resizing technique lowers the computational complexity, and essential image features are preserved. In under-sampling, the majority class helps to solve the class imbalance problem sometimes found in medical image datasets. This entails choosing, at random, a subset of the majority class samples to correspond with the minority class sample count. The under-sampling method balances the dataset, preventing the model from being biased toward the more plentiful class and enhancing the classification performance for the minority class. These preprocessing techniques guarantee the model has a well-prepared input, improving its capacity to learn discriminative features and produce accurate forecasts.

### 3.2. Feature Extraction

Feature extraction in this study was accomplished using the DenseNet121 model, a deep convolutional neural network noted for its efficient feature learning capabilities. The pre-trained DenseNet121 network automatically extracts hierarchical characteristics from the histopathological pictures. These characteristics record high-level semantic information, tumor patterns, and low-level minutiae, edges, and textures. Extracted from several DenseNet121 layers, the deep feature maps offer rich, multi-scale representations later utilized for categorization. This automated feature extraction guarantees a complete and substantial collection of features for accurate breast cancer categorization, lowering the demand for human involvement.

#### Proposed DenseNet121 Architecture

**Figure 2:**
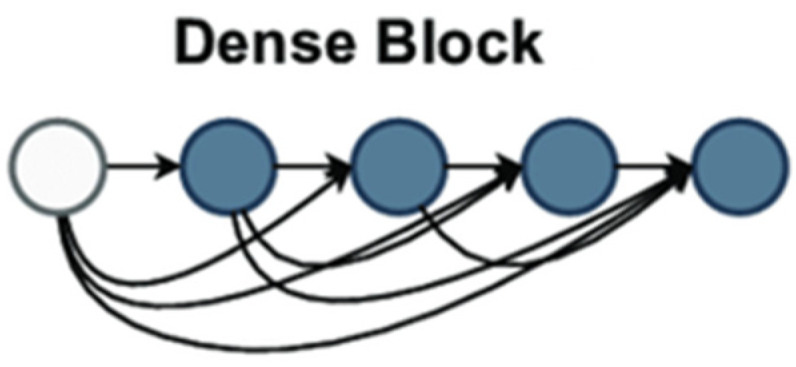
Dense block architecture used as a backbone in the proposed feature extraction module.

Introduced in their paper [35], Huang et al. described a deep learning architecture for convolutional neural networks (CNNs). Using a unique connectivity technique inside CNNs, DenseNet tackled difficulties including feature reuse, vanishing gradients, and parameter efficiency, delivering significant breakthroughs to computer vision. DenseNet links every layer in a block to all preceding levels, unlike standard CNNs, where each layer connects to the one following. This tight connectivity means every layer is input from all preceding layers, providing a more significant information flow over the network. Figure 2 demonstrates dense block architecture used as backbone in feature extraction.

The dense block provided in DenseNet121 is a fundamental design choice that increases feature reuse, improves gradient flow, and lowers overfitting in deep convolutional neural networks. Every dense block comprises numerous layers, each receiving input from all prior layers inside and from the following layer. This differs significantly from traditional CNN architectures in which each layer receives feedback from the previous one. The dense block architecture guarantees appropriate information flow and feature reuse based on dense connectivity—where each layer’s output is concatenated with that of all previous levels. In a dense block, the mathematical formulation for the input of the all-the-layer ix stated in Equations (1) and (2).

(1) $$x_{l}=F_{l}({[x}_{0},x_{1},\ldots ,x_{l-1}])$$

(2) $$x_{l}=Concatenate\left(x_{0},x_{1},\ldots ,x_{l-1}\right)$$

Concatenate signifies the concatenation operation along the channel dimension; $x_{0},x_{1},\ldots ,x_{l-1}$ are the feature maps from all preceding layers. Each layer runs a set of procedures to extract features from the concatenated input, usually convolution, batch normalization, and ReLU activation. The *l*-th layer’s convolutional operation can be written as stated in Equation (3).

(3) $$F_{l}=Conv\left(BatchNorm\left(ReLu\;\left(x\right)\right)\right)$$

*ReLU* is the rectified linear unit activation function; conv is a convolutional operation; and the batch norm is batch normalization. By addressing vanishing gradients, which are prevalent in deeper networks, this composition helps preserve the network’s stability during training. Using all the features guarantees that every layer can access the whole feature map from all preceding layers, improving gradient flow and preventing overfitting. DenseNet121’s growth rate K, the count of feature maps generated by every layer, is a fundamental idea. DenseNet121’s growth rate was set at 32. Hence, every layer added 32 fresh feature mappings to the output. This guarantees the network learns rich feature representations progressively without appreciably raising the parameter count. For example, the output at the sixth layer of a dense block will contain 32 × 6 = 192 feature maps, and there are six layers in the dense block. DenseNet models are mostly more efficient than conventional CNNs because this growth rate limits the number of parameters added per layer, therefore helping to control the complexity of the model. DenseNet121 consists of four dense blocks with varying layer counts. The first dense block contains six layers; the second has twelve; the third has twenty-four; the fourth has sixteen. While the network deepens, the number of output channels from every block increase; the total number of feature mappings increases at every block. With each next block learning even more complicated patterns, this technique lets the network capture low-level and high-level characteristics across the layers. Following their creation of the feature maps, the dense blocks undergo global average pooling to downplay the spatial dimensions while maintaining the image’s global features. This process generates a fixed-length vector for every feature map by averaging each across all spatial dimensions, height and breadth. Globally average pooling is defined mathematically as the feature map $x_{l}$ global average pooling operation, as shown in Equation (4).

(4) $$GlobAvgPool\left(x_{l}\right)=\frac{1}{H\times W}\sum_{i=1}^{H}\sum_{j=1}^{W}x_{l}\left(i,\;j\right)$$

where *H* and *W* are the height and width of the feature map and $x_{l}(i,\;j)$ denotes the value at the (i, j)-th position in the feature map. This stage dramatically reduces the computational complexity and parameter count, increasing the model’s efficiency. Finally, the output of the global average pooling layer flows via a fully connected (FC) layer that serves as the classifier for the network. The ultimately linked layer generates the final classification output using the pooled feature vector. DenseNet121’s design allows more efficient learning by enabling greater feature reuse, improving gradient flow, and lowering overfitting using its dense connection and controlled growth rate. The model is helpful for photo-classification since its design enables it to keep a limited parameter count while collecting complicated information. From the link of the dense block to the global average pooling and ultimately linked layers, every element, rich and hierarchical feature representations, is vital in ensuring the network learns.

### 3.3. Classification

A connected DNN for classification uses the obtained feature set as input. The multiple layers within the DNN are meant to interpret and improve the features for precise prediction. The obtained features first fit the dense layers since they are flattened into a one-dimensional vector. This vector then spans 512 neuron-thick layers. Batch normalization stabilizes learning, improves convergence, and reduces the overfitting risk following every dense layer. The rectified linear unit (ReLU) activation function provides non-linearity after every dense layer, enabling the network to learn complex patterns. Dropouts with a rate of 0.2 randomly turn off neurons during training, thus preventing overfit and ensuring that the model generalizes well to unprocessed input. The softmax activation function of the last output layer. This layer generates probability for every class, therefore allowing binary and multi-class classification. Under binary classification, two probability values representing benign and malignant categories define the outcome. The probability for numerous benign and malignant subtypes makes up multi-class classification. This method leverages the DenseNet121 architecture for efficient feature extraction, ensuring that both low-level and high-level features are captured. DNNs further process these features to yield accurate predictions. This pipeline achieves a robust performance by combining dense connectivity, pooling, batch normalization, dropout, and softmax activation while minimizing overfitting and computational complexity. This design is particularly suited for medical image classification tasks, where high accuracy and generalization are critical.

### 3.4. Evaluation Measures

This study utilized performance evaluation criteria to examine the pre-trained models, including accuracy, precision, recall, f1-score, and ROC. A confusion matrix is a quantitative tool used to assess the performance of a classification model. It provides a structured table that displays the correct and incorrect predictions made by the model during testing. The confusion matrix (CM) is a widely used method for evaluating the predictive accuracy of a trained model on a validation dataset. True Negatives represent the proper identification of normal, while False Negatives occur when inaccurately classified as usual.

The accuracy of a classification model is a performance metric that quantifies its overall effectiveness. The statistics are calculated by dividing the number of correctly predicted cases (True Positives and True Negatives) by the total number of instances in the dataset, as shown in Equation (5).

(5) $$Accuracy=\frac{TP+TN}{TP+FN+TN+FP}\times 100$$

Precision is a measure of how accurately a categorization model predicts favorable outcomes. The sum of True Positives and False Positives yields the total count, as shown in Equation (6).

(6) $$Precision=\frac{TP}{TP+FP}$$

Recall is a performance statistic that assesses how well a classification model captures all pertinent instances of a positive class. Some other terms for recall include sensitivity and true positive rate. It is calculated as True Positives divided by the sum of True Positives along with False Negatives, as shown in Equation (7).

(7) $$Recall=\frac{TP}{TP+FN}$$

F1-Score: The F1-score, sometimes referred to as the F1 measure, combines precision and recall into a single result in a confusion matrix, making it a valuable performance metric. It is especially beneficial when deciding whether precision or recall is more important. The F1-score is computed by employing Equation (8).

(8) $$F1\text{-}Score=\frac{2TP}{2TP+FP+FN}\times 100$$

The Receiver Operating Characteristic curve, or ROC, is a graphical plot that shows how well a classification model works at different levels for classification problems. The ROC curve is made by plotting the rate of True Positives against the rate of False Positives at each baseline level.

AUC (Area Under the Curve) represents the area under the Receiver Operating Characteristic (ROC) curve, which plots the True Positive Rate (TPR or recall) against the False Positive Rate (FPR) at various classification thresholds. The AUC metric quantifies the model’s ability to distinguish between classes, with a value of 1 indicating perfect classification and 0.5 representing random guessing. In this study, AUC was used to evaluate the model’s performance in both binary and multi-class classification tasks, providing a robust measure of its discriminative power.

## 4. Results and Discussion

This section on Results and Discussion uses the BreakHis dataset to provide a comprehensive analysis of the suggested model’s performance for binary and multi-class classification problems. Key performance measures, including accuracy, precision, recall, F1-score, and the confusion matrix, directed the assessment of the model’s ability to distinguish benign from malignant tumor types. The model’s performance was examined concerning data augmentation, class balancing, and feature extraction methods, stressing both model strengths and constraints in handling class imbalance and obtaining high classification accuracy. The results are contextualized by pertinent comparisons to current techniques, stressing the pragmatic consequences of histological image processing. The following are some key evaluations conducted in this study.

- The performance of the proposed method was evaluated for binary classification to assess its accuracy and effectiveness in distinguishing between benign and malignant cases.
- The proposed method’s performance was analyzed for multi-class classification, focusing on its ability to differentiate between various subcategories of benign and malignant conditions.
- The effectiveness of the proposed method was further validated by evaluating its performance on an alternative dataset, demonstrating its generalizability.
- A comparative analysis was performed to benchmark the proposed method’s performance against other traditional classification algorithms.
- The proposed method was compared to state-of-the-art approaches to highlight its advancements and effectiveness in solving the classification problem.

### 4.1. Dataset Preparation

As shown in Table 1, the BreakHis [36] dataset’s dataset-splitting strategy shows a well-organized method for preparing data for binary and multi-class classification chores. It addresses class imbalance via data augmentation and emphasizes the distribution of benign and malignant samples throughout training, validation, and testing sets. Guaranteeing balanced learning and accurate evaluation of machine learning models depends on this method. Four classes define the benign category: adenosis, fibroadenoma, tubular adenoma, and phyllodes tumor. There are 2480 photos in the original benign sample dataset, distributed unevenly among the classifications. With 1014 photographs, fibroadenoma is the most common class; phyllodes tumor is the least often seen class with 453 images. Data augmentation corrected this disparity by increasing the dataset size for every class, producing 7440 augmented photos overall. This augmentation technique guarantees enough representation of the minority classes, lowering possible bias during model development. The expanded dataset was then separated into 4466 photos for training, 1487 for validation, and 1487 for testing, guaranteeing a balanced representation of all benign classes in each group.

**Figure 3:**
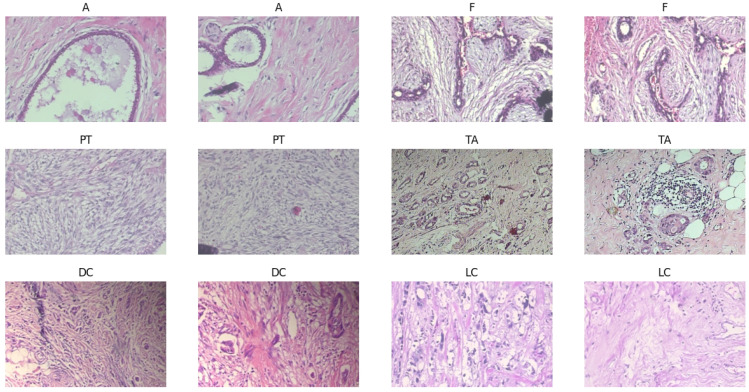

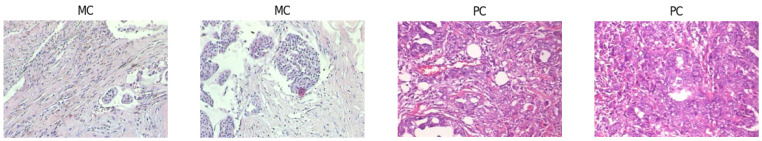
Sample images from the BreakHis v1.0 database.

Four further classes fall under the malignant category: ductal carcinoma, lobular carcinoma, mucinous carcinoma, and papillary carcinoma, as the samples shown in Figure 3. Comprising 5429 pictures, the initial malignant dataset is larger than the benign dataset. With 3451 photographs, ductal carcinoma is the most common type; papillary carcinoma has 560 images. Augmentation methods expanded the collection to 9385 photos, much like benign categories. This increase guarantees that minority classes—such as papillary carcinoma—are adequately represented in training. The cancerous dataset was split into 5631 photos for training, 1677 for validation, and 1677 for testing using the same balanced distribution technique. The balanced dataset attained via augmentation and even class presence directly affects the performance of machine learning models. This configuration guarantees the model does not favor one category for binary classification tasks (benign vs. malignant), producing more consistent predictions. The additional dataset helps the model to learn unique features for every class in multi-class classification, hence lowering the risk of misclassification—especially for minority classes such as papillary carcinoma or phyllodes tumor. Furthermore, important for evaluating real-world applicability is the objective measurement of the model’s generalizing capacity by validating and testing subsets from unseen data.

The sample pictures highlight important morphological characteristics necessary for categorization and show the histological variation of benign and malignant breast tumors. While fibroadenoma (F) combines stromal and glandular components, benign tumors, including adenosis (A), exhibit extensive glandular features. Whereas tubular adenoma (TA) shows homogeneous tubular glands inside the fibrous stroma, phyllodes tumor (PT) shows leaf-like stromal overgrowth. Among the malignant samples are lobular carcinoma (LC), marked by single-file tumor cell configurations, and ductal carcinoma (DC) with chaotic invasive patterns. Training strong models depends on this visual heterogeneity, guaranteeing correct feature extraction and classification across several cancer types.

### 4.2. Binary Classification

**Figure 4:**
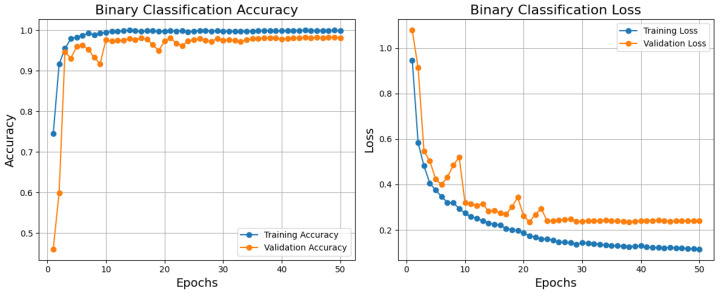
Training, validation, accuracy, and loss graphs for binary classification model trained using the BreakHis dataset.

Based on the training, validation accuracy, and loss curves, the binary classification model performs relatively well, as shown in Figure 4. Reflecting a good generalization, the training accuracy improves rapidly and stabilizes near 1.0; the validation accuracy also rises quickly and stabilizes just below the training accuracy. Comparatively, the validation loss declines initially but stabilizes somewhat higher than the training loss; the training loss declines steadily and reaches a low value. Although standard in machine learning models, this little difference between training and validation measurements points to minor overfitting, which is not shown in this paper. After about 10 epochs, both measures stabilize, suggesting that the model has converged successfully. Although the model performs poorly, one could use dropout, L2 regularization, or data augmentation to further lower overfitting. The model is generally highly accurate and has low loss, which qualifies it for practical uses where its present degree of generalization is suitable.

**Figure 5:**
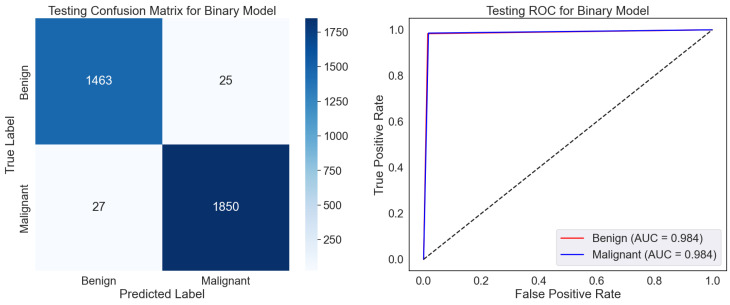
Testing confusion matrix and ROC for binary classification using the BreakHis dataset.

The confusion matrix displays the classifications of the test dataset, as shown in Figure 5. The model sufficiently classified 1493 benign and 1850 malignant samples, even though 25 were misclassified as malignant (False Positives) and 27 malignant samples as benign. High values relative to the off-diagonal (misclassifications) along the diagonal—True Positives and Negatives—showcase strong performance. The low False Positive and Negative rates hint at a model that strikes a mix between sensitivity—actual positive rate—and specificity—valid negative rate. The ROC curve gauges the ability of the model to differentiate among classes across several classification thresholds as shown in Figure 5. The Area Under the Curve (AUC) values of 0.984 for benign and malignant classes indicate a great discriminative capacity. A model having perfect two-class separation is marked by an AUC of 1.0. The proximity of the curve to the top left corner emphasizes the remarkable categorizing ability of the model even more.

The model exhibits excellent dependability and accuracy in predicting benign and cancerous samples. High AUC scores and low misclassification rates imply the model is suitably calibrated for binary classification problems. In situations where minimizing False Negatives, that is, missing malignant cases, it may be advisable to look at the few misclassifications to maximize the model’s sensitivity even more. With minimum misclassifications and great separability of the two classes, the confusion matrix and ROC curve show generally that the model is quite effective. It is appropriate for pragmatic application in tasks requiring exact binary classification.

Table 2, using the BreakHis dataset, displays the class-wise testing report for the binary classification model, highlighting its fantastic performance. Reaching 98.50%, the model shows remarkable accuracy, F1-score, recall, and precision for both benign and malignant classes. Most importantly, this reveals how effectively the model separates benign from malignant breast tumor samples for a correct diagnosis. However, the somewhat lower accuracy and recall for the benign class than the malignant cases suggest a potential predisposition toward spotting malignant samples. A natural data imbalance or minor feature variations in benign samples could cause this difference. By using techniques like data augmentation or class-specific loss modifications, resolving these shortcomings could enhance the generalization and resilience of the model even further.

### 4.3. Benign Subtype Classification

**Figure 6:**
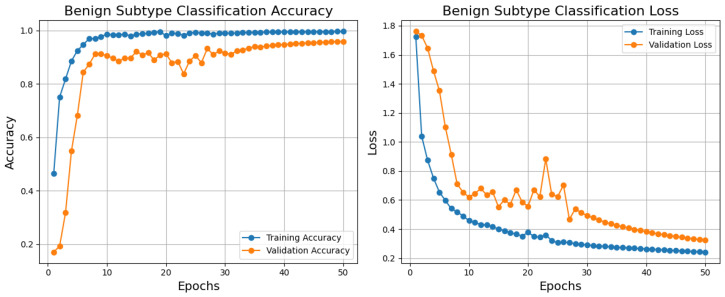
Training, validation, accuracy, and loss graphs for benign subtype classification model trained using the BreakHis dataset.

The training, validation accuracy, and loss curves for the benign subtype classification model developed on the BreakHis dataset demonstrate strong performance and pragmatic learning, as shown in Figure 6. While the validation accuracy approaches almost 0.9, showing that the model generalizes well to unknown data, the training accuracy climbs fast and stabilizes near 1.0. With some variations around epoch 20, the validation loss follows a similar trajectory but stabilizes somewhat higher; the training loss reduces continuously and stabilizes at a low amount. The little difference between training and validation measurements suggests either minimal overfitting or variability in the validation data as causes of these oscillations. The model indicates effective learning during training by converging inside the first 10–15 epochs. The applicability of this approach to benign subtype classification, necessary for histopathological research and medical diagnosis decision-making, helps to clarify why. Using low loss and outstanding accuracy, the model can enable pathologists to more regularly and successfully identify benign subtypes, thereby reducing diagnosis errors and improving workflow efficiency. Although the model shows no overfitting, its high performance suggests it can be a dependable tool for automated classification, improving clinical decision-making and patient outcomes. Enhanced validation strategies or regularizing techniques could raise its dependability even more to control oscillations and small overfit.

**Figure 7:**
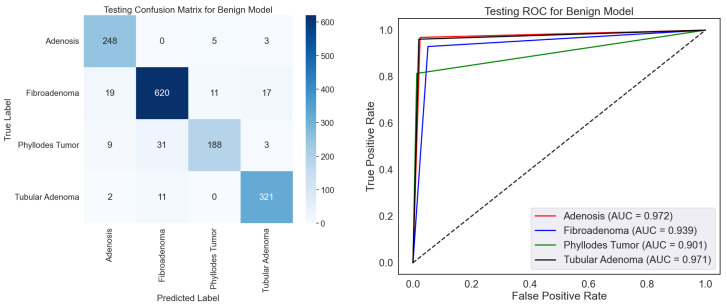
Testing confusion matrix and ROC for benign classification using the BreakHis dataset.

On the BreakHis dataset, the confusion matrix and ROC curves together fully assess the effectiveness of the benign classification model, as shown in Figure 7. The confusion matrix shows that the model can discriminate among four benign subtypes: adenosis, fibroadenoma, phyllodes tumors, and tubular adenoma. With minor misclassification, the high values along the diagonal show great ability in appropriately identifying these subtypes. Fibroadenoma, for example, shows the best correct classification count (620), while other subtypes, including adenosis (248) and tubular adenoma (321), also show good results. On occasion, nevertheless, the model mixes phyllodes tumor with fibroadenoma (31 cases), which would indicate either underlying similarity in their properties or dataset limits. The ROC curves show the discriminative power of the model with AUC values of 0.972, 0.939, 0.901, and 0.941, corresponding to adenosis, fibroadenoma, phyllodes tumor, and tubular adenoma. Especially for adenosis and tubular adenoma, these findings exhibit a remarkable capacity to distinguish the subtypes. Phyllodes tumor suggests overlapping features with other subtypes may make their categorization more challenging, given their lowest AUC of 0.901.

The capacity of this approach to automatically improve the classification of benign subtypes, crucial tasks in histopathological research, makes it relevant in testing. Correct subtype classification helps one to grasp the basic pathophysiology and change the patient care approach. The high AUC values and classification accuracy indicate that the model can be a consistent decision-support tool, lowering the pathologist’s workload and raising diagnosis accuracy. Still, further resilience by resolving misclassifications could come from more outstanding optimization via better feature extraction or data augmentation. In medical diagnosis, this concept presents rather significant pragmatic opportunities.

Table 3 shows the class-wise testing report for the benign classification model on the BreakHis dataset, together with performance measures for four benign tumor types. Reflecting its resilience, the model earns intense precision, recall, and F1-scores over most classes. Reflecting the extraordinary capacity of the model to identify this class precisely, tubular adenoma demonstrates the best overall performance with an F1-score of 94.70% and almost perfect recall (96.10%). Similarly, adenosis and fibroadenoma scored highly, particularly in recall, with values above 96% suggesting an appropriate identification of these cancer types. Phyllode tumors had a recall of 81.40% and an F1-score of 86.40%. The minimal complexity of phyllodes tumors in the sample could cause this disparity and challenge generalization. Although the model performs well, increasing the recall for phyllodes tumors could guarantee consistency among all benign categories.

### 4.4. Malignant Subtype Classification

**Figure 8:**
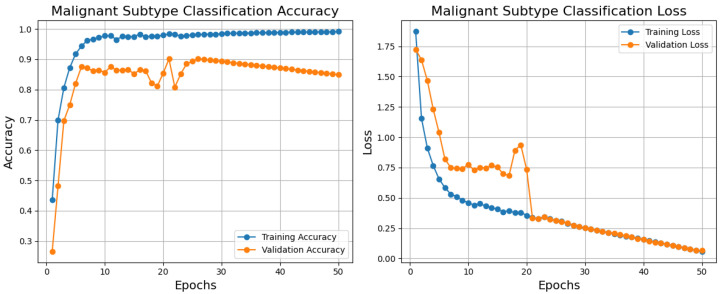
Training, validation, accuracy, and loss graphs for malignant subtype classification model trained using the BreakHis dataset.

Below are the training, validation, and loss curves for a malignant subtype classification model generated over 50 epochs using the BreakHis dataset, as shown in Figure 8. The blue training accuracy curve shows that the model effectively learns from the training data, that is, it stabilizes close to 1.0 and rises significantly in the first epochs. Though it varies between epochs 10 and 20, showing modest instability or variation in the validation set, the validation accuracy curve (orange) improves fast and reaches essentially 0.9. Given that the model effectively minimizes error on the training set, the blue training loss steadily lowers and stabilizes at a low value. The validation loss (orange) rapidly decreases in the first epoch and stabilizes somewhat higher than the training loss with little changes over training. Training and validation curve differences point to overfitting or noisy validation data as possible causes. The model generally shows excellent performance with high accuracy and low loss for training and validation sets, suggesting its ability to classify malignant subtypes. Regularizing methods, learning rate changes, or data augmentation would solve the little overfitting noted. The importance of this model stems from its ability to provide correct diagnosis and individualized treatment planning in histopathological analysis using exact and efficient malignant subtype classification.

**Figure 9:**
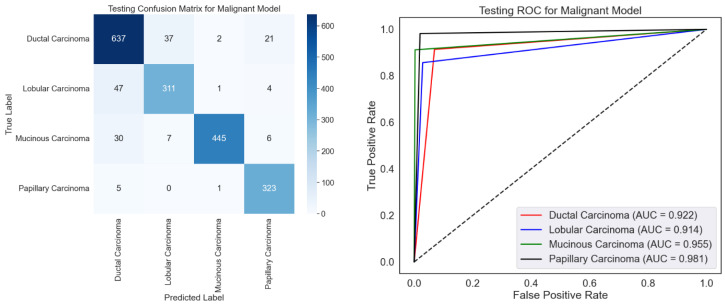
Testing confusion matrix and ROC for malignant classification using the BreakHis dataset.

ROC curves and a confusion matrix are presented to evaluate the malignancy categorization model, as shown in Figure 9. The confusion matrix performs well since it mainly recognizes most samples accurately across all malignant types. Certain misclassifications, particularly for ductal carcinoma, which have the most misclassified samples relative to other classes, show overlapping traits or imbalanced data. With high AUC values for all categories, especially papillary carcinoma (AUC = 0.981), the ROC curve emphasizes the model’s efficacy by displaying excellent discriminating capacity. In both groups with relatively lower AUC values—0.922 and 0.914, respectively—ductal and lobular carcinoma show room for development. Although excellent optimization would help solve slight misclassification tendencies, the model performs poorly.

Emphasizing several types of malignant carcinoma, Table 4 exhibits variation in the class-wise testing performance of the malignant classification model using the BreakHis dataset. The model receives the best F1-score for mucinous carcinoma (95.00%), reflecting its outstanding accuracy (99.10%) and balanced recall (91.20%). Papillary carcinoma does well with a high recall (98.20%) and a fantastic F1-score of 94.60. Having a 90.00% F1-score, mainly from a precision of 88.60%, ductal carcinoma shows somewhat low performance. Driven by its relatively weak recall (85.70%), lobular carcinoma has the lowest F1-score (86.60%), which makes it difficult to find all relevant samples. Although the model usually performs well for mucinous and papillary carcinoma, minor defects for ductal and lobular carcinoma could result from class imbalances or overlapping characteristics and demand significant change to improve performance across classes consistently.

### 4.5. Comparison with Other Classification Methods for Binary and Multi-Class Classification

Table 5 contrasts the testing performance of the proposed Deep Neural Network (DNN) model with other classification algorithms, including SVM (RBF), Random Forest (RF), XGBoost, LightGBM (LGBM), and Decision Tree (DT) across accuracy, recall, F1-score, accuracy, and AUC metrics. The suggested DNN achieves competitive precision (98.20%) and recall (98.30%), exceeding all existing models in recall and F1-score for benign classification. With the best accuracy (98.70%), recall (98.60%), and F1-score (98.60%), the DNN performs wonderfully in malignant classifications, far above XGBoost and RF. The DNN also obtains the best overall accuracy (98.50%) and AUC (0.98%), indicating better discrimination capacity and resilience than other models. Although algorithms such as XGBoost and RF also demonstrate outstanding performance, especially with somewhat higher precision for benign instances, their recall and general accuracy trail behind DNN, therefore underlining the balanced classification capacity of the DNN. The relatively lower metrics of conventional algorithms such as SVM, DT, and LGBM clearly show their flaws; these could result from their inability to adequately capture complicated patterns instead of the DNN. Exceeding all performance criteria, the suggested DNN is overall the most dependable and strong model, so it is fit for binary classification problems using BreakHis dataset.

For benign subtype classification, Table 6 shows a thorough comparison of the proposed Deep Neural Network (DNN) to present classification techniques, including SVM (RBF), Random Forest (RF), XGBoost, LightGBM (LGBM), and Decision Tree (DT). With the best accuracy (92.50%) and AUC (0.94), the proposed DNN shows extraordinary general performance, reflecting its remarkable capacity to separate across classes. For most classes, including adenosis (96.90% recollection, 92.90% F1-score) and tubular adenoma (96.10% recall, 94.70% F1-score), the DNN routinely gets the best recall and F1-score among individual subtypes. With a precision of 93.70% and a recall of 96.00%, fibroadenoma likewise beats others. The DNN underperforms in recall (81.40%) for the phyllodes tumor class, which results in a lower F1-score (86.40%) compared to RF (90.60%) and XGBoost (91.00%), signaling perhaps problems in managing this class. Class imbalances or overlapping features in a dataset could cause this problem. DNN’s general ability to outperform in precision, recall, and F1-score for most classes shows its robust performance and fit for benign subtype classification with slight exceptions for problematic subtypes such as phyllodes tumors.

For malignant subtype classification based on precision, recall, F1-score, accuracy, and AUC, the proposed Deep Neural Network (DNN) is thoroughly compared in Table 6 with other machine learning algorithms, including SVM (RBF), Random Forest (RF), XGBoost, LightGBM (LGBM), and Decision Tree (DT). Particularly in mucinous and papillary carcinoma classification, where it obtains the best precision (99.10% and 91.20%, respectively) and recall (91.20% and 98.20%, respectively), the DNN beats all other models in practically all metrics across most classes. With a stronger overall performance in managing the balance between accuracy and recall than the competing techniques, it has higher F1-scores for these classes—95.00 and 94.60.

Among all models, the DNN also obtains the highest precision (88.60%) and recall (91.40%) for ductal carcinoma, therefore generating the best F1-Score (90.00), as shown in Table 7. Likewise, for lobular carcinoma, the DNN achieves the best precision (87.60%), recall (85.70%), and F1-Score (86.60) by just slightly outperforming other approaches. Regarding general metrics, the DNN indicates strong model performance and better discriminating capacity by achieving the most fantastic accuracy (91.40%) and AUC (0.94). Although competing methods like XGBoost and LGBM exhibit quite similar performance, notably in mucinous and papillary carcinoma, their F1-scores and AUC trail behind the DNNs demonstrate their potential to learn more complex patterns. DNN’s progressively better recall in medical diagnostics highlights its capacity to reduce False Negatives—a crucial trait. Although the DNN is the best overall, the little performance difference for other classifications—such as ductal and lobular carcinoma—indicates that more optimizations could increase precision and recall in these problematic cases.

### 4.6. Comparison with State-of-the-Art Methods

Table 8 compares several feature extraction and classification methods used on the BreakHis dataset for tasks involving binary and multi-class classification. The proposed DenseNet121-based DNN obtains the best accuracy (98.50%) and AUC (0.98) for binary classification, outperforming current methods including Deep Belief Networks (98.00%, 0.97) and Ensemble CNNs (95.31%, 0.96). These results highlight the efficacy and resilience of the suggested methodology in separating benign from malignant cases. Comparable to state-of-the-art approaches, including the ViT-based method, which reached 94.80% accuracy with a somewhat higher AUC of 0.97, the suggested model achieves an accuracy of 92.50% and an AUC of 0.94 for multi-class classification. Other approaches demonstrate competitive performance with 92.96% and 94.18% accuracy, respectively: GLCM features with Self-Attention Random Forest and ViT-DeiT CNN. CNN + SVM (86.23%) and DenseNet (84.00%) show lower accuracies, implying restrictions in generalizing across classes or detecting complicated features.

Table 8 emphasizes better outcomes by combining strong classification models with excellent feature extraction. Although the proposed DenseNet121-based DNN performs exceptionally well in binary classification, it can also maximize performance in multi-class issues to surpass rival techniques such as ViT. The results highlight the importance of AUC even more as a fundamental statistic, as the dependability of the suggested model depends on its improved AUC. Restricted feature extraction or insufficient model complexity to control complex histological patterns in the BreakHis dataset could lead to issues with alternative approaches, such as DenseNet and basic CNNs. The proposed model achieves an accuracy of 92.50% and an AUC of 0.94 for multi-class classification, comparable to state-of-the-art methods, including the ViT-based method, which reached 94.80% accuracy with a somewhat higher AUC of 0.97. Other approaches demonstrate competitive performance with 92.96% and 94.18% accuracy, respectively: GLCM features with Self-Attention Random Forest and ViT-DeiT CNN. CNN + SVM (86.23%) and DenseNet (84.00%) show lower accuracies, implying restrictions in generalizing across classes or detecting complicated features.

While reaching great accuracy and AUC for binary classification, the proposed DenseNet121-based DNN shows notable constraints in multi-class classification tasks, where its performance (92.50% accuracy, 0.94 AUC) falls somewhat short of state-of-the-art approaches, like ViT (94.80% accuracy, 0.97 AUC). This suggests that the model might find it challenging to distinguish between finer-grained subclasses of malignant tissues due to the intrinsic complexity of the dataset or the lack of customization for multi-class tasks. Moreover, the computational requirements of DenseNet121, including memory and processing needs, may limit its implementation in environments with limited resources, highlighting a trade-off between model complexity and practical usability.

## 5. Conclusions

In this work, using the BreakHis dataset, we developed a DenseNet121-based Deep Neural Network (DNN) for histological breast cancer picture classification. With 98.50% accuracy and an AUC of 0.98, the suggested approach attained state-of-the-art performance for binary classification, proving its efficacy in differentiating benign from malignant instances. The model was competitive for multi-class classification with an accuracy of 92.50% and an AUC of 0.94, proving its capacity to separate numerous subtypes of malignant tumors. Comparisons of the proposed model with present methods reveal its strength in both categorization scenarios. Still, constraints in multi-class classification and computational complexity suggest chances for improvement. Our results mainly show the opportunities for deep learning-based methods to enhance computer-aided breast cancer diagnosis.

Future research will maximize the model for better performance in multi-class classification, especially in managing finer-grained distinctions among cancer subtypes, therefore addressing the limits of the proposed method. We intend to add more datasets with various imaging situations and tumor traits to improve generalizability. Furthermore, lightweight model designs will be investigated to lower computing needs, allowing deployment in areas with limited resources, such as rural healthcare settings. Additionally, the top priority will be integration with explainability methods, including saliency maps, to increase model transparency and support confidence in clinical decision-making. Lastly, broadening the research to include clinical validation and merging deep learning techniques with additional diagnostic modalities, such as genomic data, could further improve diagnosis accuracy and usefulness.

## Figures and Tables

**Table 1:** Dataset splitting of BreakHis for binary and multi-class classification.

Category	Class	Original	Augmented	Training	Validation	Testing
Benign	Adenosis	444	1332	799	267	266
	Fibroadenoma	1014	3042	1828	606	608
	Tubular Adenoma	569	1707	1024	341	342
	Phyllodes Tumor	453	1359	815	272	272
	Total	2480	7440	4466	1487	1488
Malignant	Ductal Carcinoma	3451	3451	2071	690	690
	Lobular Carcinoma	626	1878	1126	376	376
	Mucinous Carcinoma	792	2376	1426	475	475
	Papillary Carcinoma	560	1680	1008	336	336
	Total	5429	9385	5631	1877	1877

**Table 2:** Class-wise testing report for binary classification model using the BreakHis dataset.

Class	Precision	Recall	F1-Score	Accuracy
Benign	98.20%	98.30%	98.30%	98.50%
Malignant	98.70%	98.60%	98.60%	

**Table 3:** Class-wise testing report for benign classification model using the BreakHis dataset.

Class	Precision	Recall	F1-Score	Accuracy
Adenosis	89.20%	96.90%	92.90%	92.50%
Fibroadenoma	93.70%	96.00%	93.30%	
Phyllodes Tumor	92.20%	81.40%	86.40%	
Tubular Adenoma	93.30%	96.10%	94.70%	

**Table 4:** Class-wise testing report for malignant classification model using the BreakHis dataset.

Class	Precision	Recall	F1-Score	Accuracy
Ductal Carcinoma	88.60%	91.40%	90.00%	91.40%
Lobular Carcinoma	87.60%	85.70%	86.60%	
Mucinous Carcinoma	99.10%	91.20%	95.00%	
Papillary Carcinoma	91.20%	98.20%	94.60%	

**Table 5:** Comparison of Testing Performance of Proposed DNN with other Classification Algorithms for Binary Classification.

Class	Measure	SVM (RBF)	RF	XGBOOST	LGBM	DT	Proposed (DNN)
Benign	Precision	97.21%	98.36%	99.02%	98.51%	98.33%	98.20%
	Recall	94.20%	93.64%	95.10%	93.84%	92.57%	98.30%
	F1-Score	96.51%	96.10%	97.05%	95.21%	95.66%	98.30%
Malignant	Precision	92.36%	91.03%	95.25%	90.53%	91.20%	98.70%
	Recall	96.81%	98.50%	98.47%	98.63%	97.80%	98.60%
	F1-Score	94.52%	94.08%	95.37%	94.56%	93.78%	98.60%
Accuracy		95.73%	95.81%	96.09%	95.89%	94.40%	98.50%
AUC		0.95	0.95	0.96	0.95	0.94	0.98

**Table 6:** Comparison of testing performance of the proposed DNN with other classification algorithms for benign subtype classification.

Class	Measure	SVM (RBF)	RF	XGBOOST	LGBM	DT	Proposed (DNN)
Adenosis	Precision	88.50%	89.10%	88.80%	89.00%	87.50%	89.20%
	Recall	94.50%	95.00%	95.50%	94.80%	94.20%	96.90%
	F1-Score	91.50%	92.00%	92.10%	91.70%	90.40%	92.90%
Fibroadenoma	Precision	91.50%	92.10%	91.60%	92.20%	90.30%	93.70%
	Recall	94.00%	95.50%	95.30%	94.80%	94.20%	96.00%
	F1-Score	92.70%	93.80%	93.40%	93.50%	91.20%	93.30%
Phyllodes Tumor	Precision	89.00%	90.20%	89.60%	89.50%	88.20%	92.20%
	Recall	90.50%	91.00%	92.40%	91.80%	90.30%	81.40%
	F1-Score	89.70%	90.60%	91.00%	90.60%	89.10%	86.40%
Tubular Adenoma	Precision	92.00%	92.60%	92.20%	92.00%	91.30%	93.30%
	Recall	94.40%	95.30%	94.90%	94.50%	93.80%	96.10%
	F1-Score	93.20%	93.90%	93.60%	93.20%	92.50%	94.70%
Accuracy		91.60%	92.10%	91.90%	91.90%	91.00%	92.50%
AUC		0.92	0.93	0.93	0.93	0.91	0.94

**Table 7:** Comparison of testing performance of the proposed DNN with other classification algorithms for malignant subtype classification.

Class	Measure	SVM (RBF)	RF	XGBOOST	LGBM	DT	Proposed (DNN)
Ductal Carcinoma	Precision	87.50%	88.10%	88.20%	88.00%	87.80%	88.60%
	Recall	90.00%	90.50%	91.00%	90.20%	89.80%	91.40%
	F1-Score	88.72%	89.29%	90.09%	89.08%	88.78%	90.00%
Lobular Carcinoma	Precision	86.50%	87.00%	87.30%	87.10%	86.80%	87.60%
	Recall	84.50%	85.00%	85.50%	85.20%	84.80%	85.70%
	F1-Score	85.49%	86.00%	86.39%	86.13%	85.79%	86.60%
Mucinous Carcinoma	Precision	97.50%	98.00%	98.50%	98.00%	97.80%	99.10%
	Recall	90.00%	90.50%	91.00%	90.80%	90.60%	91.20%
	F1-Score	93.61%	94.01%	94.65%	94.07%	93.96%	95.00%
Papillary Carcinoma	Precision	90.10%	90.50%	91.00%	90.60%	90.40%	91.20%
	Recall	97.00%	97.30%	97.80%	97.40%	97.20%	98.20%
	F1-Score	93.38%	93.68%	94.27%	93.77%	93.57%	94.60%
Accuracy		90.00%	90.50%	91.00%	90.60%	90.40%	91.40%
AUC		0.90	0.91	0.89	0.91	0.90	0.94

**Table 8:** Performance comparison with existing methods using the BreakHis dataset.

Ref. Year	Feature Extraction Method	Classification Method	Classification (Binary/Multi-Class)	Accuracy	AUC
[37], 2023	Deep Features (ResNet18)	KNN	Binary	94.20%	0.94
		SVM	Binary	97.40%	0.97
		DT	Binary	91.00%	0.90
[38], 2024	CNN	CNN with SVM	Binary	86.23%	0.86
[39], 2024	DenseNet	DenseNet	Binary	84.00%	0.84
[40], 2023	ViT-DeiT	CNN	Multi-Class	94.18%	0.94
[41], 2024	CNN	CNN	Binary	94.00%	0.94
[19], 2024	DenseNet201	DenseNet201	Binary	91.37%	0.90
[42], 2023	GLCM Features	Self-Attention Random Forest	Multi-Class	92.96%	0.95
[43], 2024	Ensemble CNN	Ensemble CNN	Binary	95.31%	0.96
[44], 2024	Auto Encoders for Features	ViT	Binary	96.50%	0.96
		CCT	Binary	97.81%	0.97
		Mobile ViT	Binary	94.33%	0.94
		ViT	Multi-Class	94.80%	0.97
		CCT	Multi-Class	84.63%	0.84
		Mobile ViT	Multi-Class	87.84%	0.88
[45], 2024	DBF	Deep Belief Network (DBF)	Binary	98.00%	0.97
-	DenseNet121	Proposed DNN	Binary	98.50%	0.98
			Multi-Class	92.50%	0.94

## Data Availability

The data used in this research are publicly available at: https://web.inf.ufpr.br/vri/databases/breast-cancer-histopathological-database-breakhis/ (accessed on 12 January 2025).
